# Type of Care and Living Situation Are Associated with Nutritional Care but Not Nutritional Status of Older Persons Receiving Home Care

**DOI:** 10.3390/healthcare8030296

**Published:** 2020-08-25

**Authors:** Neshat Chareh, Eva Kiesswetter, Anja Rappl, Peter Stehle, Helmut Heseker, Cornel C. Sieber, Dorothee Volkert

**Affiliations:** 1 Institute for Biomedicine of Aging, Friedrich-Alexander-Universität Erlangen-Nürnberg, Kobergerstrasse 60, 90408 Nuremberg, Germany; eva.kiesswetter@fau.de (E.K.); cornel.sieber@fau.de (C.C.S.); dorothee.volkert@fau.de (D.V.); 2 Institute for Medical Informatics, Biometry, and Epidemiology, Friedrich-Alexander-Universität Erlangen-Nürnberg, Universitätsstrasse 22, 91054 Erlangen, Germany; anja.rappl@fau.de; 3 IEL-Nutritional Physiology, Rheinische Friedrich-Wilhelms-Universität Bonn, Nußallee 9, 53115 Bonn, Germany; p.stehle@uni-bonn.de; 4 Institute of Nutrition, Consumption and Health, Universität Paderborn, Warburgerstrasse 100, 33098 Paderborn, Germany; helmut.heseker@upb.de; 5 Department of Medicine, Kantonsspital Winterthur, Brauerstrasse 15, 8400 Winterthur, Switzerland

**Keywords:** type of care, living situation, nutritional care, nutritional status, older adults

## Abstract

Nutritional care and nutritional status may differ in older persons receiving informal (IC) or professional (PC) home care and further depend on the living situation, but little is known in this regard. In this analysis of a cross-sectional multicenter study, type of care, living situation, and nutritional care were enquired in 353 older adults (≥65) receiving IC or PC, living either with partner (LP), with others (LO) or alone (LA), and the nutritional status was determined by BMI and MNA^®^. For IC receivers, food shopping (IC-LP 94%, IC-LO 96%, IC-LA 92%) and warm meals (IC-LP 89%, IC-LO 90%, IC-LA 71%) were mainly provided by relatives, whereas 47% of PC-LA prepared warm meals by themselves and 22% received meals on wheels. Thirteen percent were underweight, 13% malnourished, and 57% at risk of malnutrition without differences between the groups. Adjusted odds ratios (OR) of being malnourished were also not different (IC-LP 2.2 [95% CI 0.5–9.7], IC-LO 1.4 [0.3–6.6], IC-LA 1.4 [0.3–6.6]) compared to PC-LA. In conclusion, provision of nutritional care obviously differed according to the type of care and living situation, whereas nutritional status does not seem to be affected by these aspects. More research is clearly needed in this field.

## 1. Introduction

With a global increase of old and very old people, who are more often frail and functionally impaired, a greater demand for care and support is expected [1]. Most older adults are willing to spend their last years of life in their own household [2]. Previous studies in several European countries have shown that more than half of the older adults receiving care at home are living alone [3,4,5,6].

Older adults receiving care at home, may receive care from professional caregivers and/or informal caregivers with whom they have a social connection such as spouses, children, close friends, and neighbours [7,8]. Home-care assistance from professional caregivers encompasses various types of services to meet the older adults’ needs, ranging from basic housekeeping needs such as household cleaning and shopping to medical care and highly technical infusion therapies [9]. In Germany, older persons living at home and being in need of care receive services from obligatory public nursing care insurance either in cash, when informal caregivers are available and are able to organize the care by themselves, or in kind in the form of professional care services [10]. A combination of both types of services is also possible [11].

Older persons who live at home and are in need of care, are at increased risk of malnutrition; prevalence rates up to 35% have been reported in this population [3,12,13,14,15]. Multiple factors may contribute to this increased risk. On the one hand, individual factors, especially impaired functional and health status—the main reasons for care dependency—are important determinants of malnutrition [16]. Care dependency may go along with limited ability to perform daily activities such as shopping, preparing meals, and eating, which might constrain food choices and intake and thus contribute to the development of malnutrition [16]. On the other hand, social aspects and quality of care, in particular quality of nutritional care, affect availability of food, food intake, and nutritional status [16]. It has been reported that older adults living alone or having fewer social relations are more susceptible to develop malnutrition than those having better social networks [16,17,18]. The consequences of malnutrition in older persons are serious, including reduced quality of life, increased health care costs, higher risk of being hospitalized for longer time and a higher risk to die earlier [13,15,19,20].

Providing adequate nutritional care, i.e., support with shopping, preparing meals, eating and drinking, for this frail group seems to be crucial, but may be required in varying degrees, depending on the overall need of care, and may be provided by different people and services. It has been shown in a study on older adults living at home and being in need of care in Sweden that those who did not live alone received more nutritional care from their informal caregivers compared to those who lived alone [6]. Another study also from Sweden similarly reported that informal caregivers are the primary source of providing nutritional care and support in older adults living in private households [21]. Information about the need and provision of nutritional care for older persons in Germany in different living situations—alone or with others—and receiving different types of care—informal or professional—is not available. It is also not yet evaluated whether living situation and type of care may influence the nutritional status of older adults cared for at home. With regard to the increasing preference of older people to stay at their own household as well as changing family structures, which may reduce the availability of informal care in the future, provision of adequate nutritional care might become a future public health challenge [22]. Information about nutritional care provision in different living arrangements and with different types of care of older persons receiving home care is thus important and may support planning of future services ensuring adequate nutritional care. In this regard, the present work aims to examine the association of type of care and living situation first with nutritional care and second with the nutritional status of older adults receiving home care in Germany.

## 2. Materials and Methods

### 2.1. Study Design and Recruitment

For the present analyses, existing data from the ErnSIPP study (‘Ernährungssituation von Seniorinnen und Senioren mit Pflegebedarf in Privathaushalten’, ErnSIPP) [23,24] were used. In this cross-sectional multicentre study, 353 community-dwelling older adults were recruited in three German cities (Bonn, Paderborn, and Nuremberg) between March and December 2010 with the help of the medical service of the health insurance companies in Germany (MDK), home care providers, care counselling offices, and day care institutions. Inclusion criteria were age 65 years and older, living in a private household, no terminal illness, and a care level according to the German nursing insurance system. The care level indicates a need for daily nursing care or help with housekeeping of at least 90 min and was categorized into three levels: Care level I means 90 ≤ 180 min of care per day, care level II 180 ≤ 300 min/day, and care level III ≥ 300 min/day [25].

A domiciliary visit took place by a trained nutritionist following an agreement via a phone call, and participants’ characteristics, including living situation and type of care, nutritional care, and nutritional status were assessed by standardized questionnaires in personal interviews. In the case of cognitive impairment (Mini Mental State Examination < 17 points), primary caregivers were asked to complete the questions (except those regarding depressive mood and satisfaction with nutritional care). Body weight and height were measured according to standardized procedures [26]. The ethics committees of the Friedrich-Alexander-Universität Erlangen-Nürnberg (Nr. 4122), Germany, and the Rheinische Friedrich-Wilhelms-Universität Bonn (Nr. 271/09), Germany approved the study protocol. Each participant or his/her legal custodian signed the written informed consent form.

### 2.2. Participants’ Characteristics

Participants’ age, gender, care level, appetite, presence of dementia, chewing and swallowing problems, frequency of receiving visits, number of chronic diseases, and number of presently prescribed drugs were recorded in standardized questionnaires. Mini Mental State Examination (MMSE; 0–30 points) and Geriatric Depression Scale (GDS; 0–15 points), were used to assess the cognitive and emotional status of participants, respectively [27,28]. The ability to perform basic activities of daily living (ADL; 0–100) was assessed according to Mahoney and Barthel [29]. A score below 35 points was defined as severe limitations, 35–60 points as moderate limitations, and 65–100 points as slight limitations [30].

### 2.3. Living Situation and Type of Care

Participants were asked, with whom they live currently (with partner, with children, with children and partner, with others or alone). Those who lived with children, children and partner or with others were combined into one group as, living with others. To define the type of care, participants were asked whether they receive informal care, professional care or both. For the analyses, the answers were dichotomized in “mainly informal” and “mainly professional” care. In participants who received both informal and professional care, living situation and the estimated weekly time provided for care were used to decide about group allocation. Participants who lived together with their informal caregiver were always assigned to the group receiving mainly informal care. Participants who did not live with their informal caregivers were allocated to the more extensive type of care.

Based on this information about living situation and type of care, participants were categorized into four groups: Participants who received mainly informal care living with their partner (IC-LP), participants who received mainly informal care living with others (IC-LO), participants who received mainly informal care living alone (IC-LA), and participants who received professional care living alone (PC-LA). As only six participants who received mainly professional care but also informal care, did not live alone, and the sample size to build a respective separate group was too small, these participants were allocated to the groups IC-LP (*n* = 5) or IC-LO (*n* = 1).

### 2.4. Nutritional Care

Regarding nutritional care, participants were asked, who mainly does the shopping for food and how is the person being cared for provided with warm meals. Multiple responses were possible including: Self, relatives, friends, care service, and meals on wheels. Furthermore, need of help with eating (i.e., with chopping foods; having the spoon raised to the mouth; verbal prompting to eat: Yes/no) and with drinking (i.e., with opening the bottle; having a cup raised to the mouth; verbal prompting to drink: Yes/no) as well as satisfaction with current nutritional care (very satisfied/satisfied/not very satisfied) were asked.

### 2.5. Nutritional Status

Nutritional status was determined by the Mini Nutritional Assessment (MNA; 0–30 points) and by Body Mass Index (BMI) [31,32]. Based on the total MNA score, participants were categorized as well nourished (>23.5 points), at risk of malnutrition (17–23.5 points) or malnourished (<17 points) [31]. BMI was calculated as weight (kg)/height (m^2^). According to the Global Leadership Initiative on Malnutrition (GLIM), low BMI was defined with the cutoff values of <20 kg/m^2^ for adults 65–69 years and <22 kg/m^2^ for adults ≥70 years [32]. Normal BMI was defined as 20 ≤ 30 kg/m^2^ for age 65–69 years and 22 ≤ 30 kg/m^2^ for age ≥70 years, and high BMI as ≥30 kg/m^2^ independent of age.

### 2.6. Data Aanalysis

Results from categorical data are presented as percentage and from continuous data as median (P25–P75). The combined variable “type of care and living situation” was related to participants’ characteristics, nutritional care, and nutritional status by applying the chi-square test followed by the z-test with Bonferroni correction for categorical data and Kruskal-Wallis test followed by all pairwise comparisons with Bonferroni correction for continuous data. Participants with missing values were not considered in statistical analyses. Furthermore, where appropriate Fisher’s exact test was applied. Two multinomial regression models were estimated to examine the effect of “type of care and living situation” on the nutritional status, measured as MNA, one unadjusted and the second adjusted for age, gender, dementia, swallowing problems, and ADL, where age was considered as continuous variable, gender, dementia, and swallowing problems as dichotomous variables and ADL as ordinal variable. Similarly, two logistic regression models were calculated with low BMI as the outcome variable. In the models, participants who received professional care and lived alone (PC-LA) were considered as the reference group. A *p*-value <0.05 was considered as a statistically significant result. Statistical analysis was performed using the SPSS version 25.0 (IBM, Munich, Germany).

## 3. Results

### 3.1. Participants’ Characteristics

Participants’ median age was 82.0 years, and 63.7% were female. In addition, 86.1% received mainly informal care, and 59.2% did not live alone, among them 67.5% living with a partner and 32.5% with others, mainly children. Based on type of care and living situation, 39.9% received informal care and lived with their partner (IC-LP), 19.3% received informal care and lived with others (IC-LO), 26.9% received informal care and lived alone (IC-LA), and 13.9% received professional care and lived alone (PC-LA) (Table 1).

Those living with a partner were significantly younger (79.0 years) and more often male (57.4%) than those living with others or alone. Among participants who received informal care, those who lived alone suffered less often from severe limitations in ADL than those who did not live alone, and accordingly only a few of them were assigned to care level III. Participants with IC-LO suffered most often from dementia (54.4%) and showed less often normal/slightly impaired cognition based on MMSE. More than 70% of the participants in IC-LO reported to have chewing problems and more than 34% of participants who received informal care and did not live alone suffered from swallowing problems. Those participants who lived alone received significantly more often daily visits compared to those who did not live alone. Participants in the four groups did not significantly differ concerning appetite, depressive mood, number of chronic diseases, and number of daily drugs.

### 3.2. Nutritional Care

**Figure 1:**
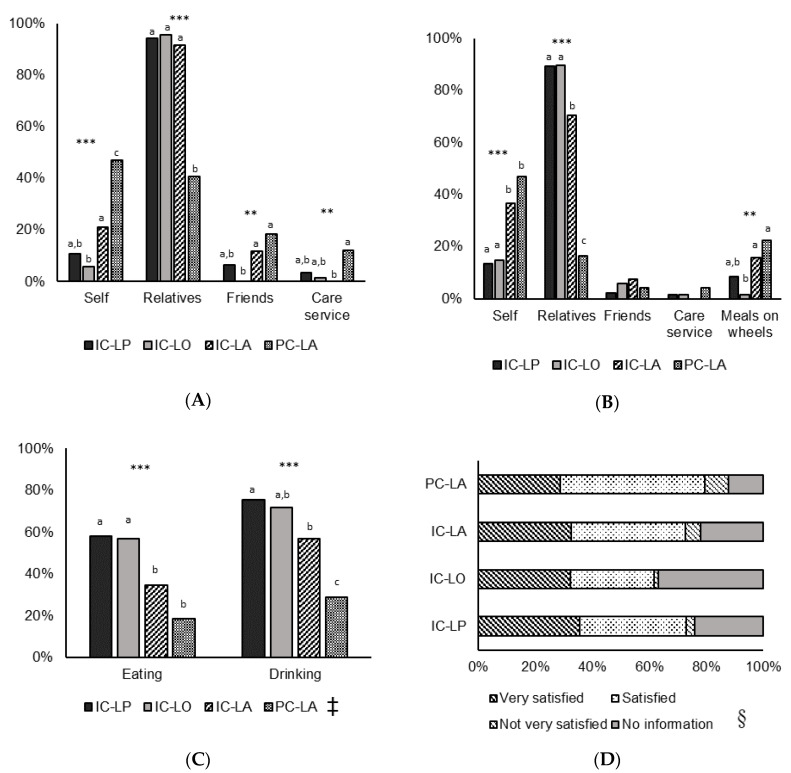
Nutritional Care and Satisfaction with Nutritional Care According to Living Situation and Type of Care. IC-LP (*n* = 141): Informal Care-Living with Partner; IC-LO (*n* = 68): Informal Care-Living with Others; IC-LA (*n* = 95): Informal Care-Living Alone; PC-LA (*n* = 49): Professional Care-Living Alone; (**A**) who goes shopping?; (**B**) who provides warm meals?; (**C**) need of help with eating and drinking; (**D**) satisfaction with nutritional care; differences between groups: Chi-square test followed by z-test with Bonferroni correction; *** *p* < 0.001, ** *p* < 0.01; a, b, c percentage values with unlike superscript letters were significantly different in post hoc tests; ^‡^ *n* = 352; ^§^ *p* value = 0.484.

In PC-LA, 46.9% of those went shopping on their own compared to 10.6% in IC-LP, 5.9% in IC-LO, and 21.1% in IC-LA (Figure 1A). In contrast, 94.3%, 95.6%, and 91.6% of food shopping in IC-LP, IC-LO, and IC-LA was provided by relatives, respectively, compared to 40.8% in PC-LA (Figure 1A). In addition, 46.9% of PC-LA and 36.8% of IC-LA were cooking on their own compared to 13.5% of IC-LA and 14.7% of IC-LO (Figure 1B). In contrast, 89.4%, 89.7%, and 70.5% of warm meals for IC-LP, IC-LO, and IC-LA were provided by relatives, respectively, compared to 16.3% in PC-LA (Figure 1B). Overall, 46.0% and 63.1% of participants were in need of help with eating and drinking, respectively. Participants who did not live alone were more often in need of help with eating (about 60%) and drinking (about 70%) than those living alone. Participants in PC-LA were least often in need of help (Figure 1C). Satisfaction with nutritional care did not significantly differ between the four groups, however, a large proportion of missing values needs to be taken into account (24.4% of all) (Figure 1D).

### 3.3. Nutritional Status

In total, 13.3% of participants were malnourished, and 57.2% were at risk of malnutrition. The median for BMI was 27.4 kg/m^2^. Participants who received professional care and lived alone showed a higher median of BMI and accordingly a higher prevalence of BMI ≥ 30, although the differences were not statistically significant (Table 2).

Both the results of the unadjusted and adjusted multinomial models demonstrate a nominal decrease of odds ratios in the overall majority of cases after adjustment; none of the results, however, was significant (Table 3). When nutritional status was measured as BMI, similar to MNA, none of the effects of interest was significant. In contrast to malnutrition according to MNA, odds ratios for low BMI were overall reduced in those receiving IC compared to PC-LA.

## 4. Discussion

In this cross-sectional data analysis of older persons receiving care at home in Germany, marked differences in providing nutritional care with regard to food shopping and preparing warm meals were observed between participants living with others or living alone and receiving informal care or professional care. However, no association between living arrangement and type of care and nutritional status was found.

In addition to these main results, a remarkable finding was the relatively high percentage of informal care and low percentage of participants living alone. More than 85% of the older adults in our study received mainly informal care, which is higher, compared to the general home care situation in Germany in 2011, where 67% of all people receiving care at home received only care from informal caregivers [33]. This marked difference might be explained by the fact that our participants did not receive only informal care. The proportion of informal care in our study is also higher compared to a large Swedish study, which found about 70% of older adults receiving care at home being cared for by informal caregivers [21]. Regardless of the type of care, approximately 41% of all participants in our study lived alone, which is relatively low compared to previous research showing a range of 53% to 74% of older adults receiving care at home and living alone [3,5,6,34]. One possible explanation for this high percentage of informal care and the low percentage of living alone might be a lack of interest to participate in our study among those older adults receiving professional care or living alone. Furthermore, these older adults may be too frail to be able to participate in the study. Additionally, from the perspective of professional careers in the case of being responsible for answering the questionnaires, taking part in our study might have been too time-consuming and an unfeasible extra burden for them.

In agreement with other studies on older adults cared for at home, participants in our study were also characterized by high age, multimorbidity, and polypharmacy (Table 1) [4,8]. Furthermore, the distribution of care levels in our study sample was in line with the general home care situation in Germany (care level I 59% vs. 62%; care level II 30% vs. 30%; care level III: 11% vs. 8%) [33].

Nutritional care provision in our study was markedly higher in those participants who received informal care and did not live alone, which was in line with their overall need of care and also in principal agreement with previous findings [6,21]. Purchasing food was mainly carried out by relatives for subjects receiving informal care (Figure 1A). This is a very plausible result, given the small additional effort for caregivers to buy just a bit more when they are shopping for themselves, and was similarly reported in a study on care-dependent older adults living at home in Sweden where over 90% of food provision for those who did not live alone and 60% for those who lived alone was managed from informal caregivers such as partner and children [6]. Similar to food shopping, those older adults who received informal care also received their warm meals mainly from relatives, in contrast to those receiving professional care (Figure 1B). In our study, participants who lived alone were significantly less dependent in basic activities of daily living, which may explain the higher proportion of those who go shopping and prepare their warm meals by themselves compared to those who did not live alone. Furthermore, in line with our study, it has been reported that older adults living at home and being cared for by informal as well as professional caregivers are more dependent compared to those older adults with professional care only [21]. While professional care staff covered services regarding personal activity of daily living in this study, informal careers assisted with instrumental activity such as shopping and cooking [21].

Only 4.1% of older adults receiving professional care received warm meals from their care service, and more than 20% of them made use of meals on wheels, a service which delivers prepared meals for those older persons who are not able to do it by their own [4] (Figure 1B). Limited time during each visit is one of the main barriers for professional care staff to engage in nutritional care, which is especially relevant for those older adults in need of help with eating and drinking [15,35,36]. However, it cannot be distinguished in our study if the small contribution of care services in nutritional care is because of the limited time or the better physical functioning of these participants.

Although in our study, no significant difference was observed between the four groups concerning satisfaction with nutritional care, it might be recognized that those receiving professional care more often reported being less satisfied compared to those receiving informal care (Figure 1D). However, due to the small sample of participants who received professional care and also the large proportion of missing values overall because of cognitive impairment and inability to report nutritional satisfaction reliably, these findings must be interpreted with caution and further study is needed to evaluate the association between nutritional care and nutritional satisfaction.

Prevalence of malnutrition (13.4%) and risk of malnutrition (57.4%) were slightly higher compared to a meta-analysis from Cereda et al. [37], which reported 8.7% and 47.5% of older adults cared for at home being malnourished or at risk of malnutrition, respectively, also based on MNA. The lacking association between the prevalence of malnutrition and living arrangement and type of care was surprising. It has been reported that social conditions such as living alone might be associated with the risk of malnutrition [38]. Results in this regard were however, conflicting, and it seems essential to differentiate between living alone and feeling alone in this population group. Living alone does not necessarily lead to malnutrition, but it has been demonstrated that the feeling of loneliness is associated with an increased risk of malnutrition [38,39]. Although unfortunately no question addressed the feeling of loneliness in our study, more than 85% of older adults living alone reported to receive daily visits, which can be interpreted as being socially active.

Moreover, nutritional status is the result of inadequate dietary intake, we further analyzed the association of energy and protein intake based on three-day food records with type of care and living situation. In agreement with nutritional status also nutritional intake was similar in the four groups, except of a higher energy intake in female participants who received professional care compared to those females who received informal care and lived with their partners (Appendix A Table A1). In addition to these findings regarding absolute intake, it is important to notice that around 60% and 50% of participants could not meet their actual energy and protein requirements, again similar proportions in all four groups (Appendix A Table A2).

An important factor affecting nutritional status of older adults receiving care at home might be the level of nutritional knowledge of the caregivers. It has been shown that a six months nutritional education intervention for caregivers (informal as well as professional) of older persons cared for at home and at risk of malnutrition can improve the older adults’ nutritional status within one year [40]. In our study, professional caregivers were asked whether they received education about nutrition or not. Unfortunately, due to a very low participation rate of professional caregivers in this part of the survey, this important aspect could not be evaluated.

Albeit no significant differences in nutritional status, neither in the unadjusted nor in the adjusted model, those participants who did not live alone and received informal care had higher odds of having malnutrition compared to those who lived alone and received professional care (Model 1 in Table 3). Care dependency, presence of dementia, and also swallowing problems have been reported to contribute to an increased risk of malnutrition among older adults [15,41]. By considering these risk factors in our model, the odds decreased (Model 2 in Table 3), as the prevalence of dementia, swallowing problem, and care dependency tended to be higher among those participants who did not live alone and received informal care compared to those who lived alone and received professional care.

Furthermore, eating dependency and drinking dependency are potential determinants, which might negatively affect food intake and lead to malnutrition in older adults [16]. In our study, those who received informal care and did not live alone were more in need of help regarding eating and drinking compared to those who received professional care and lived alone. However, we did not consider these potential determinants in our model, as dependency on eating and drinking might be the consequences of dementia and diminished functional ability, which are considered in the model [42].

The study sample showed a high level of BMI (27.4 kg/m^2^), and, surprisingly, nearly half of PC-LA were obese (BMI ≥ 30 kg/m^2^), compared to 28% in those who did not live alone. In contrast to MNA, participants who received informal care mainly showed lower odds of having low BMI compared to those who lived alone and received professional care. This may be explained by the fact that MNA considers relevant risk factors leading to malnutrition in addition to parameters reflecting malnutrition. Furthermore, it might be explained by the tendency toward a lower prevalence of malnutrition-related risk factors such as dementia, care dependency, and being in need of help with eating and drinking among those who lived alone and received professional care [15,41].

### Strengths and Limitations

One of the strengths of this study is the availability of information regarding provision of nutritional care and nutritional and health status which was obtained by standardized questionnaires and geriatric assessment tools. Furthermore, this study is the first and still only study about nutritional care and nutritional status in Germany. It has a relatively large sample of older persons receiving care at home who are difficult to recruit. However, only a small group of older adults receiving professional care could be recruited which cannot be considered as representative and limited statistical analysis. In addition, only few participants receiving mainly professional care were living with others and could not be analyzed separately. The time provided for care by informal caregivers was only roughly estimated per week, with a potential risk of misjudgment. Furthermore, the data were assessed already in 2010, however, relevant changes in the caring system did not occur meanwhile.

## 5. Conclusions

This study highlighted the important topic of nutritional care and nutritional health. It demonstrates that the provision of nutritional care in German older adults receiving care at home differed according to the type of care and living situation whereas nutritional status was not associated with these aspects. Regarding the high prevalence of (risk of) malnutrition overall, informal as well as professional caregivers need to pay attention to ensure adequate nutrition regardless of the living situation of older persons in need of care. Information regarding the importance of nutrition in older adults and adequate nutritional care strategies, e.g., by specific training courses or information campaigns, could be helpful in this regard. Further, more detailed research, e.g., considering also the time needed for nutritional care and adequacy of nutritional care, in representative samples is required to better understand the effects of different living situations and types of care on nutritional status of older adults receiving home care, and to support planning of services required to ensure adequate nutritional care in the future.

## Figures and Tables

**Table 1:** Participants’ Characteristics of the Whole Sample and Stratified by Living Situation and Type of Care.

Variables	Categories	All (*n* = 353)	IC-LP (*n* = 141)	IC-LO (*n* = 68)	IC-LA (*n* = 95)	PC-LA (*n* = 49)	*p*
Female (%)		63.7	42.6 ^a^	72.1 ^b^	84.2 ^b^	73.5 ^b^	<0.001
Age median (P25–P75)		82.0 (76.0–87.0)	79.0 (72.0–83.0) ^a^	84.0 (78.0–89.0) ^b^	84.0 (80.0–89) ^b^	83.0 (78.0–87.75) ^b^	<0.001
Care level (%)	I	58.9	25.5 ^a,b^	44.1 ^b^	72.6 ^c^	71.4 ^a,c^	<0.001
	II	29.8	33.3 ^a^	33.8 ^a^	25.3 ^a^	22.4 ^a^	
	III	11.3	14.2 ^a^	22.1 ^a^	2.1 ^b^	6.1 ^a,b^	
ADL (%)	Slight limitation (65–100)	60.6	51.1 ^a^	45.6 ^a^	81.1 ^b^	69.4 ^a,b^	<0.001
	Moderate limitation (35–60)	19.0	22.7 ^a^	23.5 ^a^	10.5 ^a^	18.4 ^a^	
	Severe limitation (0–30)	20.4	26.2 ^a^	30.9 ^a^	8.4 ^b^	12.2 ^a,b^	
Cognition MMSE (%)	Normal/slightly impaired 24–30	57.5	56.0 ^a^	32.4 ^b^	65.3 ^a,c^	81.6 ^c^	<0.001
	Moderately impaired 17–23	19.5	19.9 ^a,b^	33.8 ^b^	15.8 ^a^	6.1 ^a^	
	Severely impaired 0–16	17.0	17.7 ^a^	25.0 ^a^	13.7 ^a^	10.2 ^a^	
	Missing values	5.9	6.4	8.8	5.3	2.0	
Dementia (%)		34.6	33.3 ^a^	54.4 ^b^	28.4 ^a^	22.4 ^a^	<0.001
Depressive mood GDS (%)	Normal 0–5	44.2	37.6	39.7	49.5	59.2	0.272
	Moderate 6–10	27.2	31.2	19.1	28.4	24.5	
	Severe 11–15	4.8	6.4	5.9	2.1	4.1	
	Missing values	23.8	24.8	35.3	20.0	12.2	
Number of diseases median (P25–P75)		5.0 (4.0–7.0)	5.0 (3.0–6.25)	5.0 (4.0–6.0)	5.0 (4.0–7.0)	6.0 (4.0–6.75)	0.623
Number of medicines median (P25–P75) (*n*)		7.0 (5.0–10.0) (352)	7.0 (5.0–10.0) (141)	6.0 (4.0–9.0) (68)	7.0 (5.0–9.0) (94)	9.0 (5.0–10.0) (49)	0.110
Chewing problems (%) (*n*)		52.1(351)	43.3(141) ^a^	71.6(67) ^b^	51.1(94) ^a,b^	53.1(49) ^a,b^	<0.01
Swallowing problems (%) (*n*)		28.5(351)	34.8(141) ^a^	38.8(67) ^a^	18.1(94) ^b^	16.3(49) ^a,b^	<0.01
Appetite (%)	Very good	15.6	19.9	16.2	14.7	4.1	0.211
	Good	76.8	72.3	75.0	77.9	89.8	
	Bad	7.1	7.8	5.9	7.4	6.1	
	Missing values	0.6	0.0	2.9	0.0	0.0	
Number of visits (%)	Up to 2×/ month	7.9	10.6 ^a,b^	17.6 ^b^	0^c^	2.0 ^a,c^	<0.001
	Up to 4×/ week	22.9	31.2 ^a^	27.9 ^a,b^	13.7 ^b^	10.2 ^b^	
	Daily	68.8	57.4 ^a^	54.4 ^a^	86.3 ^b^	87.8 ^b^	
	Missing values	0.3	0.7	0.0	0.0	0.0	

IC-LP: Informal Care-Living with Partner; IC-LO: Informal Care- Living with Others; IC-LA: Informal Care-Living Alone; PC-LA: Professional Care-Living Alone; Care level I: 90 ≤ 180 min care/day; care level II: 180 ≤ 300 min care/day; care level III: ≥300 min care/day; ADL: Activity of Daily Living; MMSE: Mini Mental State Examination; GDS: Geriatric Depression Scale; Differences between groups: Chi-square test followed by z-test with Bonferroni correction for categorical data, Kruskal-Wallis test followed by all pairwise comparisons with Bonferroni correction for continuous data; a, b, c median or percentage values within a row with unlike superscript letters were significantly different in post hoc tests.

**Table 2:** Participants’ Nutritional Status According to Living Situation and Type of Care.

Variables	Categories	All *n* = 353	IC-LP *n* = 141	IC-LO *n* = 68	IC-LA *n* = 95	PC-LA *n* = 49	*p*
MNA (%)	Well-nourished	29.2	23.4	29.4	35.8	32.7	0.073
	Risk of malnutrition	57.2	61.0	48.5	55.8	61.2	
	Malnutrition	13.3	15.6	20.6	8.4	6.1	
	Missing value	0.3	0.0	1.5	0.0	0.0	
BMI (%)	Low	13.3	10.6	17.6	12.6	16.3	0.051
	Normal	51.0	60.3	50.0	46.3	34.7	
	High	32.3	27.0	27.9	35.8	46.9	
	Missing value	3.4	2.1	4.4	5.3	2.0	
BMI Median (P25-P75) (n)		27.4 (24.0–31.2) (341)	27.4 (24.2–30.4) (138)	27.3 (22.9–30.5) (65)	27.0 (24.4–31.2) (90)	29.1 (24.1–33.4) (48)	0.415

IC-LP: Informal Care-Living with Partner; IC-LO: Informal Care-Living with Others; IC-LA: Informal Care-Living Alone; PC-LA: Professional Care-Living Alone; MNA: Mini Nutritional Assessment; BMI: Body Mass Index; Low BMI, <20 kg/m^2^ (65–69 years) and <22 kg/m^2^ (≥70 years); Normal BMI, 20–29.99 kg/m^2^ (65–69 years) and 22-29.99 kg/m^2^ (≥70 years); High BMI, ≥30 kg/m^2^ (≥65 years); differences between groups: Chi-square test for categorical data and Kruskal-Wallis test for continuous data.

**Table 3:** Odds of Being Malnourished or at Risk of Malnutrition and Having Low BMI According to Living Situation and Type of Care.

Variables	Categories	Model 1 (unad.)	Model 2 (ad.)
		OR (95% CI)	OR (95% CI)
MNA			
Malnutrition (*n* = 47)	PC-LA	1.00	1.00
	IC-LP	3.6 (0.9–13.7)	2.2 (0.5–9.7)
	IC-LO	3.7 (0.9–15.3)	1.4 (0.3–6.6)
	IC-LA	1.2 (0.3–5.4)	1.4 (0.3–6.6)
Risk of malnutrition Model 1 (*n* = 202) Model 2 (*n* = 201)	PC-LA	1.00	1.00
	IC-LP	1.4 (0.7–2.9)	1.0 (0.4–2.3)
	IC-LO	0.9 (0.4–2.0)	0.5 (0.2–1.3)
	IC-LA	0.8 (0.4–1.7)	0.9 (0.4–2.0)
BMI			
Low BMI (*n* = 47)	PC-LA	1.00	1.00
	IC-LP	0.6 (0.2–1.5)	0.5 (0.2–1.3)
	IC-LO	1.1 (0.4–3.0)	0.7 (0.2–2.0)
	IC-LA	0.8 (0.3–2.0)	0.8 (0.3–2.2)

IC-LP: Informal Care-Living with Partner; IC-LO: Informal Care- Living with Others; IC-LA: Informal Care-Living Alone; PC-LA: Professional Care-Living Alone; MNA: Mini Nutritional Assessment; BMI: Body Mass Index; Low BMI, <20 kg/m^2^ (65–69 years) and <22 kg/m^2^ (≥70 years); PC-LA: Reference group; Model 2 was adjusted for age, gender, ADL (activity of daily living), dementia, and swallowing problems.

## Appendix A

**Table A1:** Participants’ Energy and Protein Intake According to Living Situation and Type of Care.

Variables	Categories	All	IC-LP	IC-LO	IC-LA	PC-LA	*p*
Energy intake [kcal/d] median (P25-P75) (*n*)	Male	2016 (1605–2239) (123)	1985 (1613–2301) (77)	2086 (1771–2193) (19)	2023 (1441–2094) (15)	2000 (1651–2363) (12)	0.705
	Female	1707 (1476–1918) (216)	1542 ^a^ (1330–1818) (59)	1656 ^a,b^ (1315–1879) (48)	1745 ^a,b^ (1535–2037) (75)	1746 ^b^ (1573–1948) (34)	0.007
Protein intake [g/kg BW] median (P25–P75) (*n*)	Male	0.95 (0.80–1.21) (119)	0.95 (0.79–1.26) (75)	1.03 (0.87–1.22) (18)	0.96 (0.76–1.25) (14)	0.84 (0.74–1.04) (12)	0.242
	Female	1.00 (0.79–1.21) (209)	0.89 (0.67–1.18) (58)	0.96 (0.74–1.22) (46)	1.03 (0.89–1.22) (72)	1.00 (0.80–1.26) (33)	0.054

IC-LP: Informal Care-Living with Partner; IC-LO: Informal Care-Living with Others; IC-LA: Informal Care-Living Alone; PC-LA: Professional Care-Living Alone; energy and protein intake based on three-day food records; BW: Body weight; differences between groups: Kruskal-Wallis test; a, b, median values within a row with unlike superscript letters were significantly different in post hoc tests.

**Table A2:** Proportion of Participants Not Meeting Recommendations for Energy and Protein (%).

Variables	All *n* = 328	IC-LP *n* = 133	IC-LO *n* = 64	IC-LA *n* = 86	PC-LA *n* = 45	*p*
Energy	60.1	61.7	57.8	59.3	60.0	0.961
Protein	50.6	56.4	50.0	41.9	51.1	0.219

IC-LP: Informal Care-Living with Partner; IC-LO: Informal Care-Living with Others; IC-LA: Informal Care-Living Alone; PC-LA: Professional Care-Living Alone; each participant’s energy requirement was calculated using the individual basal metabolic rate (BMR) and physical activity level (PAL); BMR: Calculated according to gender, age, and body weight (BW); for male >60 years: BMR (kcal/d) = 13.5 × BW (kg) + 487; and for female >60 years: BMR (kcal/d) = 10.5 × BW (kg) + 596; protein requirement: 1 g/kg body weight per day; BW: Body weight; differences between groups: Chi-square test.
